# The promise and pitfalls of synteny in phylogenomics

**DOI:** 10.1371/journal.pbio.3002632

**Published:** 2024-05-20

**Authors:** Jacob L. Steenwyk, Nicole King

**Affiliations:** 1 Howard Hughes Medical Institute, University of California, Berkeley, California, United States of America; 2 Department of Molecular and Cell Biology, University of California, Berkeley, California, United States of America

## Abstract

Reconstructing the tree of life remains a central goal in biology. Early methods, which relied on small numbers of morphological or genetic characters, often yielded conflicting evolutionary histories, undermining confidence in the results. Investigations based on phylogenomics, which use hundreds to thousands of loci for phylogenetic inquiry, have provided a clearer picture of life’s history, but certain branches remain problematic. To resolve difficult nodes on the tree of life, 2 recent studies tested the utility of synteny, the conserved collinearity of orthologous genetic loci in 2 or more organisms, for phylogenetics. Synteny exhibits compelling phylogenomic potential while also raising new challenges. This Essay identifies and discusses specific opportunities and challenges that bear on the value of synteny data and other rare genomic changes for phylogenomic studies. Synteny-based analyses of highly contiguous genome assemblies mark a new chapter in the phylogenomic era and the quest to reconstruct the tree of life.

## Abstract 2

Reconstructing the tree of life is a central goal in biology. This Essay discusses the use of synteny, an emerging phylogenomic marker, for reconstructing the tree of life and propose a roadmap for conducting rigorous and accurate synteny-based phylogenomic analysis.

## Introduction

Arguably, the most ambitious goal in phylogenetics is to reconstruct the entire tree of life. To build phylogenetic trees, diverse data types have been used, and our understanding of the tree of life has undergone significant transformations with each methodological advance.

**Fig 1:**
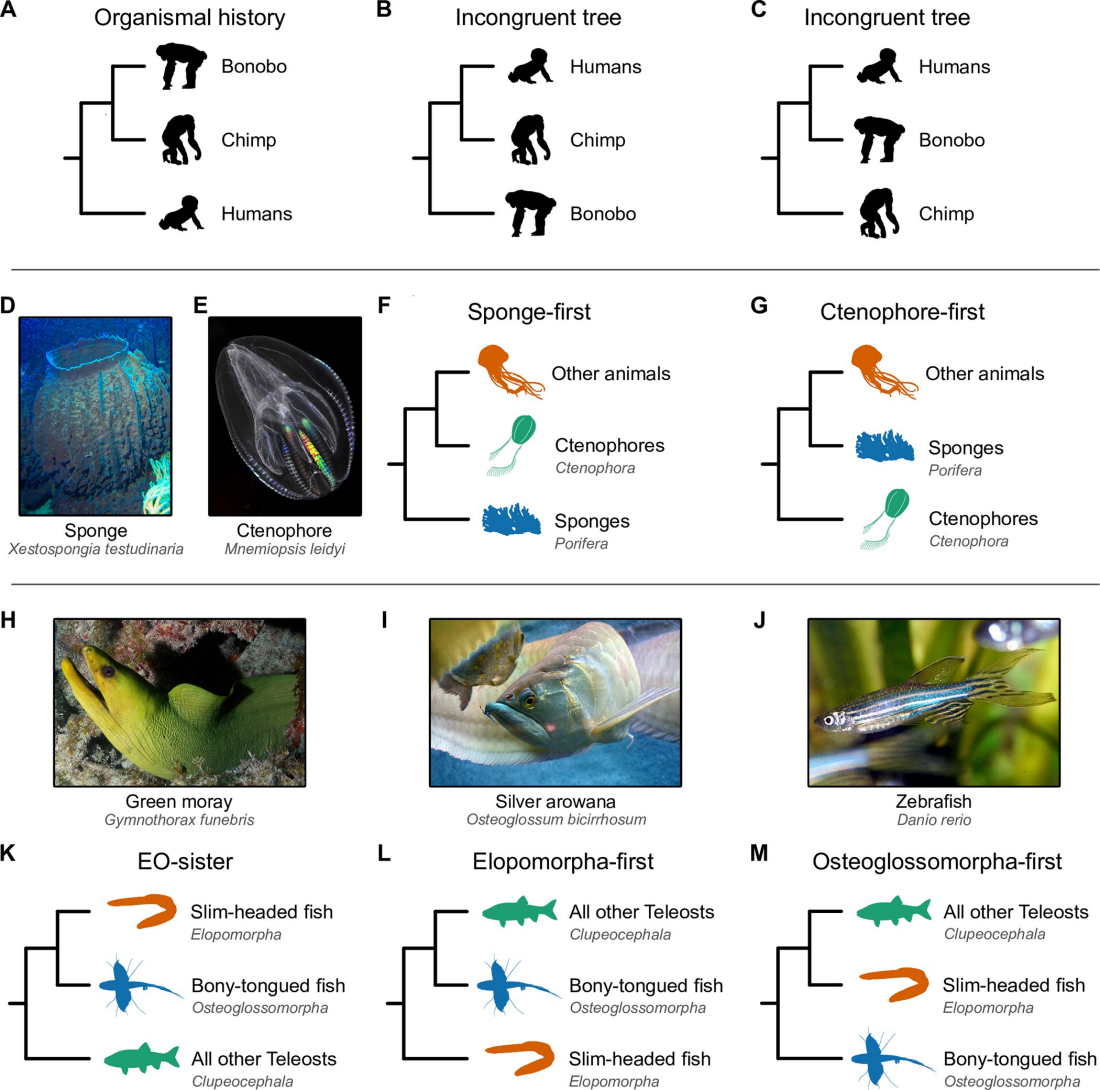
Depictions of incongruence and alternate hypotheses for primates, the base of the animal tree, and teleost fish phylogenies. (**A**) Example of tree incongruence. The weight of evidence strongly supports a sister relationship between bonobos and chimps, to the exclusion of humans. (**B**, **C**) Phylogenies that are incongruent would suggest a sister relationship between humans and chimps (**B**) or humans and bonobos (**C**). (**D**-**G**) The debate concerning early animal evolution has largely focused on whether sponges (**D**) or ctenophores (**E**) diverged first from all other animals: the sponge-first (**F**) and ctenophore-first (**G**) hypotheses, respectively. (**H**-**M**) Among teleost fish, the debate centers on the relationships among 3 major lineages—the Elopomorpha (mostly slim-headed fish; **H**), Osteoglossomorpha (mostly bony-tongued fish; **I**), and Clupeocephala (all other teleost fish; **J**). The Eloposteoglossocephala (EO-sister) hypothesis (**K**) suggests a sister relationship between slim-headed and bony-tongued fish, whereas the Elopomorpha-first (**L**) and Osteoglossomorpha-first (**M**) hypotheses suggest that slim-headed fish or bony-tongued fish, respectively, diverged before the other lineages split from one another. Recent studies that employed synteny as a phylogenomic marker supported the ctenophore-first (**G**) and EO-sister (**K**) hypotheses [8,9]. All images were obtained from the Wikimedia Commons (https://commons.wikimedia.org) or PhyloPic (https://www.phylopic.org) and are dedicated to the public domain; all credit goes to their respective contributors.

Early studies relied on aligning single or few loci to reconstruct evolutionary histories [1], but analyses of different loci often yielded phylogenies with conflicting or poorly supported topologies [2,3] (Fig 1A–1C). For example, analyses of different loci have suggested different relationships among humans, bonobos, and chimps, and among sponges, ctenophores, and bilaterians [4,5]. Numerous processes can contribute to loci with evolutionary histories that appear distinct from those of the organisms in which they are found [3,6,7], including horizontal gene transfer, convergent evolution, and incomplete lineage sorting.

The advent of cost-effective whole genome sequencing has paved the way for the phylogenomics era, in which hundreds to thousands of orthologous loci are analyzed in a total evidence approach [10,11]. The promise of phylogenomics has been that the increase in sequence data might allow phylogenetic signal to outcompete noise. Indeed, phylogenomics has successfully been used to delineate previously problematic branches within the tree of life, for example, the monophyletic grouping of nematodes and arthropods within Ecdysozoa [12,13], the placement of turtles as sister to archosaurs (crocodiles and birds) [14], and the placement of eukaryotes within Archaea [15]. These successes have positioned phylogenomics as the current standard for reconstructing most evolutionary histories. However, many branches in the tree of life remain unresolved, including those that concern key evolutionary episodes.

To address unresolved branches, phylogeneticists have sought to identify new genomic characters that accurately reflect evolutionary history, in part because they are unlikely to evolve independently in unrelated groups of organisms [16–18]. To this end, 2 recent studies [8,9] have tested the utility of gene synteny as a character for phylogenetics (Box 1). In this Essay, we review the challenges that inspired these studies, evaluate the current utility of gene synteny as a character for phylogenetics, and offer a roadmap for future use of gene synteny to reconstruct the tree of life.

**Glossary**

***Horizontal gene transfer***

Exchange of genetic material between organisms through non-reproductive mechanisms

***Convergent evolution***

The independent evolution of similar features in unrelated species

***Incomplete lineage sorting***

The retention and random sorting of ancestral polymorphisms, which can cause phylogenies based on these polymorphisms to, at times, differ from the organismal history

***Rare genomic changes***

Polymorphisms—indels, transposon integrations, changes in gene order, gene duplications, and others—excluding substitutions

***Synteny***

The conservation of the same order of loci on chromosomes from different species

***Orthology inference***

The process of determining which genes in different species are orthologs, meaning they diverged due to a speciation event

***Microsynteny***

Conservation of small blocks of genes (typically only a handful) that are found in the same order within the genome

***Macrosynteny***

Large-scale conservation of blocks of genes (hundreds to thousands or more) on chromosomes between species

***Reciprocal best BLAST hits***

A method used to find orthologous genes, in which 2 genes from different species are each other’s best match in a BLAST search

***Acrocentric chromosomes***

Chromosomes with a centromere near one end, resulting in 1 very short and 1 very long arm

***Robertsonian translocation***

A chromosomal rearrangement in which 2 acrocentric chromosomes have fused to form a single chromosome

***Taxon sampling***

The selection of taxa for a phylogenetic study

***Maximum likelihood framework***

A statistical approach used to infer evolutionary trees by finding the tree topology with the best probability given the underlying data and a model of sequence evolution

***Long-branch attraction***

An error in phylogenetic inference wherein lineages on long branches (i.e., having many substitutions per site in a data matrix) are incorrectly inferred to be closely related

***Tandem duplication***

A type of mutation in which a region of a chromosome is duplicated and the copies remain adjacent to each other

***Syntenic coverage***

The percentage of the full genome that contains syntenic blocks that are conserved in comparator genomes. Determined by taking the sum length of syntenic blocks divided by genome size

***Pangenomes***

The entire set of genes present within all strains of a species, not just those in a single reference genome

***Treeness***

A signal-to-noise measure based on the proportion of branch lengths observed among internal branches compared to internal and terminal branches

***Rogue taxa***

Taxa with placements that are unstable across a set of trees

**Ohnologs**

Genes duplicated through a whole-genome duplication event

### Tangled branches in the tree of life

There are many unresolved branches in the tree of life. Here, we focus on 2 major challenges: how to root the tree of animals, and how major clades of teleost fish, a group encompassing nearly half of all vertebrates, are related. These evolutionary questions exemplify how genome-scale data analyses can yield incongruent phylogenies and undermine our ability to fully reconstruct the tree of life.

The controversy surrounding the root of the animal tree was somewhat unexpected, as morphological comparisons had, for decades, consistently favored the placement of sponges (Fig 1D), not ctenophores (Fig 1E), as the earliest-branching lineage [19,20]; this hypothesis garnered nearly universal support during the single-locus era of phylogenetics [19,21–23] (Fig 1F). The dawn of phylogenomics, however, changed the situation. A 2008 study based on 150 genes from 77 taxa, including 2 sponges and 2 ctenophores, provided the first support for placing ctenophores at the root of the animal tree (Fig 1G) [24]. Then, in 2009, the sponge-first hypothesis was supported by a study using 128 genes and 55 taxa, including 9 sponges and 3 ctenophores [25]. Since then, investigations powered by ever larger datasets (including dozens of ctenophores and sponges) and analyzed using the latest methods in phylogenomics have provided compelling and contradictory evidence for the 2 competing hypotheses [5,25–30].

Early branching patterns in the teleost fish phylogeny are also intensely debated. Teleosts encompass 3 major clades: Elopomorpha (mostly slim-headed fish like bonefish, eels, and skipjacks; Fig 1H); Osteoglossomorpha (mostly bony-tongued fish like elephantnose fish, doublesash butterflyfish, and mormyrids; Fig 1I); and Clupeocephala (the remaining extant teleosts like pufferfish and sticklebacks; Fig 1J). Phylogenetics of some single-locus data suggested a sister relationship between Elopomorpha and Osteoglossomorpha—the Eloposteoglossocephala (EO-sister) hypothesis—in which the slim-headed and bony-tongued fish are thought to form a sister clade relative to all other teleosts [31]. However, all possible topologies (Fig 1K–1M) have received support in the phylogenomic era. Challenged by a history of conflict, some have suggested that the base of the teleost fish phylogeny is one of the most important unresolved questions in ray-finned fish evolution [32].

### Rare genomic changes as phylogenomic markers

Amid these and other ongoing debates, the value of alternative phylogenetic markers, such as rare genomic changes, has been explored [33]. Rare genomic changes are an independent source of phylogenetic information compared to primary sequence data and can complement sequence data or be used to evaluate alternative phylogenetic scenarios when sequence data are inconclusive [33]. The phylogenetic distributions of some rare genomic changes, including insertions and deletions, gene duplications and losses, and alternative genetic codes, often mirror the inferred evolutionary relationships among major vertebrate, insect, fungal, and related lineages [34–37].

The earliest studies underscoring the promise of rare genomic changes for phylogenetics were conducted before widely available whole-genome sequences. In studies conducted in the 1930s, Sturtevant and Dobzhansky reconstructed phylogenetic relationships among populations of *Drosophila pseudoobscura* by analyzing chromosomal inversions detected in the polytene chromosomes of salivary glands [38,39]. These observations led Sturtevant and Dobzhansky to suggest that comparing "different gene arrangements in the same chromosome may, in certain cases, throw light on the historical relationships of these structures, and consequently on the history of the species as a whole." Supporting this hypothesis, Hampton Carson conducted a similar analysis in 1983 to reconstruct the evolutionary relationships among Hawaiian *Drosophila* [40].

Several other cases of rare genomic changes recapitulating phylogeny have been identified. Copy number variants (duplicated or deleted loci), gene presence–absence polymorphisms, and transposable element insertions and deletions can mirror population structure and deeper-scale evolutionary relationships [41–49]. For example, lineage-specific gene duplication and loss events have been detected in humans [50] and in lineages of the bipolar budding yeast *Hanseniaspora* [37]. Genetic recoding of CUG to alanine and serine, rather than leucine, occurred in a monophyletic lineage of yeast [51]. Among more ancient divergences, the root of the angiosperm phylogeny has been successfully examined using duplication patterns of phytochrome genes [52,53].

Nonetheless, rare genomic changes are not irreproachable for phylogenetic inference. For example, rare genomic changes can evolve convergently. Losses of gene duplicates have occurred repeatedly in flatworms [54] and genetic recoding of the CUG codon from leucine to serine in Saccharomycotina fungi occurred on 2 occasions independently [55]. Convergence has also been observed among structural genomic features. For example, distributions of mitochondrial genome size, structure, and content have converged among Placozoa, chytrid fungi, and choanoflagellates [56], leading briefly to the inference that Placozoa diverged from all other animals first—a hypothesis largely refuted by phylogenomic analyses of nuclear genes [24–30,57]. Even in closely related species of walnuts, phylogenies inferred from large amounts of local gene-order data, DNA sequence alignments, and gene-family content, yield differing tree topologies [58].

Thus, the utility of rare genomic changes has been mixed. Several examples demonstrate that rare genomic changes can recapitulate evolutionary history, while others contradict generally accepted evolutionary relationships established using other data types. Determining when and what rare genomic changes should be used has been hindered by the sparsity of methods for detecting rare genomic changes and algorithms for analyzing their informativeness.

### Synteny emerges in the phylogenomic era

**Fig 2:**
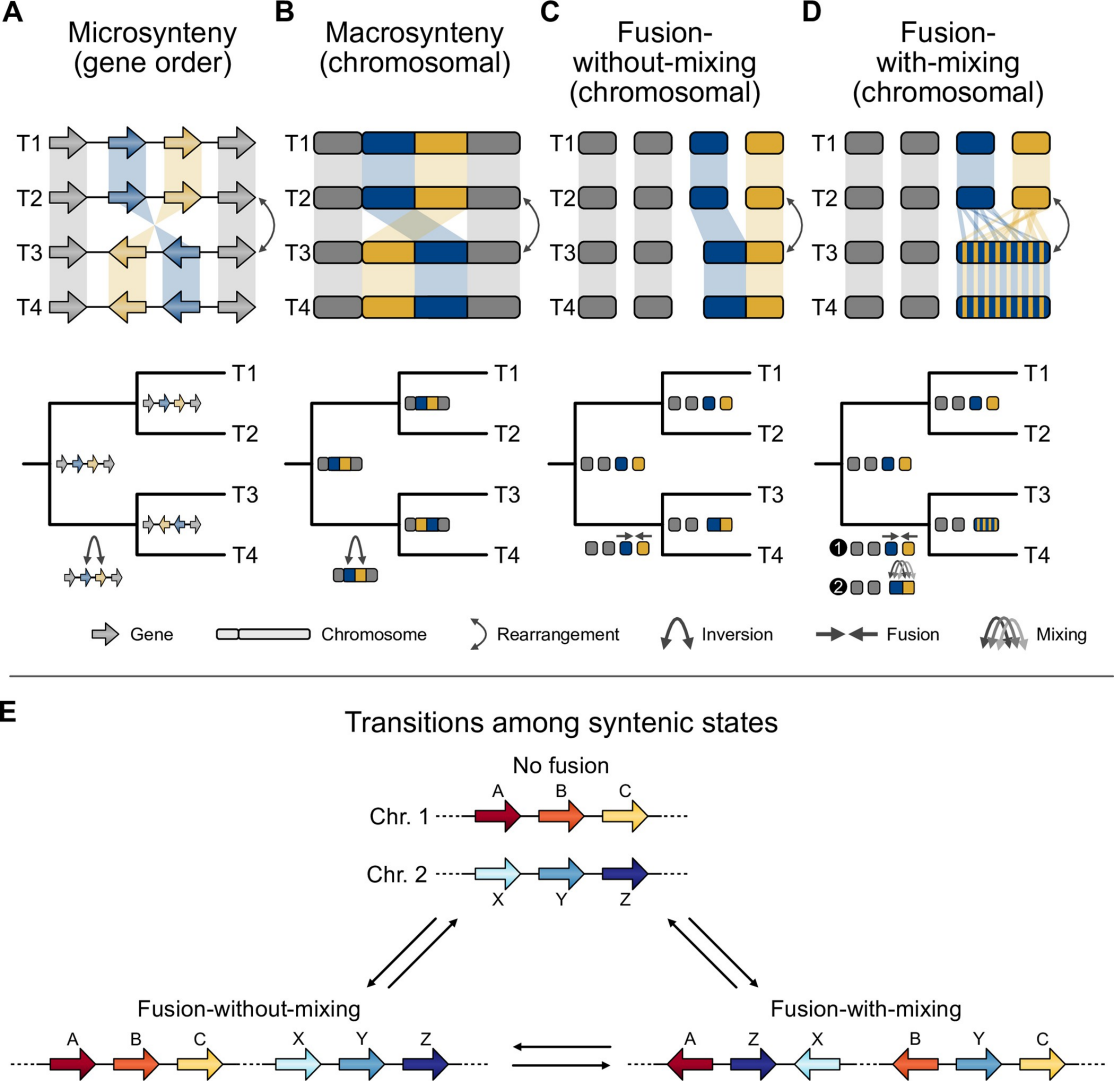
Data types for sequence-based phylogenetics. Consider the relationships among 4 taxa (represented as T1, T2, T3, and T4), wherein the pairs T1 and T2, and T3 and T4 are sister to one another. Changes in genome architecture can be examined at the scale of microsynteny (short stretches of orthologous loci; **A**) or macrosynteny (long stretches of orthologous loci; **B**). Changes in synteny can be described by different processes, such as fusion events without-mixing (**C**) and with-mixing (**D**). (**A**) In the case of microsynteny, evidence of an inversion may occur between the blue and orange loci (bottom), which happened in the ancestor of T3 and T4. (**B**) The same phenomenon can happen in the case of macrosynteny. (**C**) Fusion-without-mixing events between 2 chromosomes may also reflect phylogeny. In this case, a fusion event may have occurred in the ancestor between T3 and T4 (bottom). (**D**) Fusion-with-mixing can also be used to reconstruct phylogeny. Note, the evolutionary scenarios at the bottom of panels **A**-**D** depict only the most likely of many possible scenarios. (**E**) Fusion-with-mixing events may occur in 2 steps. First, there is a fusion event, then rearrangements occur, scrambling the order of genes that once were encoded on separate chromosomes. As a result, the probability of going from a “no fusion” to “fusion-without-mixing” state (and vice versa), and going from a “fusion-without-mixing” state to a “fusion-with-mixing” state, is relatively higher than going from a “fusion-with-mixing” to a “fusion-without-mixing” state. Transitioning directly from a “no fusion” to a “fusion-with-mixing” state is highly unlikely and may require an intermediate “fusion-without-mixing” state. Transition probabilities may vary depending on the underlying genome biology of the organism, the size of the syntenic region, and other parameters.

As abundant genome assemblies have become available and algorithm development has followed suit, the field of phylogenomics has become primed to revisit the value of rare genomic changes—specifically synteny—for phylogenetic inference. User-friendly software has enabled the detection of collinear DNA sequences in genomes from related organisms [59–64], thereby streamlining robust orthology inference [10] and analyses of changes in microsynteny and macrosynteny (Fig 2A and 2B). Shared rearrangements in gene order would be predicted to indicate a common evolutionary history, so long as convergence is not at play.

A major molecular mechanism driving syntenic variation is unequal homologous recombination [65]. Genomes with multiple copies of similar sequences, such as transposable elements in plant genomes, can be particularly prone to unequal homologous recombination [66]. Similarly, recombination between highly similar but nonallelic sequences (nonhomologous recombination) can result in major mutational events, such as recurrent deletions or duplications [67]. Other error-prone DNA repair mechanisms—including nonhomologous end joining—can also result in syntenic changes [68]. Whether a recombination event results in a microsyntenic or macrosyntenic change depends on the spacing between recombinant regions.

Saccharomycotina yeast have been a model lineage for developing and testing phylogenetic methods [69–71]. Comparison of the relationships among shared syntenic blocks in Saccharomycotina yeast with an evolutionary history previously inferred using concatenated multiple sequence alignments revealed that nearly 99% of microsyntenic blocks were more likely to be shared among closely related species than expected by random chance [72], reinforcing the notion that synteny can reflect phylogeny [73]. Subsequent developments in software and bioinformatic pipelines, vetted through simulations and examinations of empirical data, have facilitated the inference of organismal histories based on syntenic blocks [74–76]. Although promising, these studies primarily focused on establishing the utility of synteny through proof-of-principle approaches (i.e., reevaluating well-established relationships or using simulated scenarios). Applying these methods to address challenging tree of life debates has been a more recent development.

### Synteny brings fresh perspectives to the tree of life

#### Synteny and the root of the animal tree

A recent reconstruction of ancient gene linkages has brought new data to bear on the sponge-first versus ctenophore-first debate at the base of the animal tree of life [9] (Fig 1F and 1G). This study relied on a new ensemble of genome assemblies from select sponges, ctenophores, bilaterians, cnidarians, and 3 outgroup taxa—a choanoflagellate (*Salpingoeca rosetta*), a filasterean (*Capsaspora owczarzaki*), and an ichthyosporean (*Creolimax fragrantissima*). Although detecting synteny among these genomes was complicated by the accumulation of chromosomal rearrangements across deep time, comparative analyses identified syntenic blocks conserved between outgroup and animal taxa using 3-way or 4-way reciprocal best BLAST hits; 29 and 20 different syntenic blocks were shared between animals and the filasterean or choanoflagellate, respectively. Notably, all 20 syntenic regions identified in the choanoflagellate were also present in the filasterean.

The inferred evolutionary changes to otherwise conserved syntenic blocks were placed in 1 of 3 categories based on outgroup taxa—no fusion, fusion-without-mixing, and fusion-with-mixing (Fig 2C and 2D)—which were then encoded and utilized in a phylogenetic framework. “No fusion” referred to syntenic blocks that remain on separate chromosomes. For example, imagine that an ancestral organism contains genes A, B, and C on 1 chromosome and genes X, Y, and Z on another (Fig 2E). If these blocks are on separate chromosomes (chromosomes 1 and 2) in 2 descendent organisms, there was “no fusion.” In the case of “fusion-without-mixing,” syntenic blocks A and B now coexist on the same chromosome in a descendent genome compared to the ancestor. This phenomenon is relatively well documented among acrocentric chromosomes in humans, which can fuse via a Robertsonian translocation [77]. Finally, “fusion-with-mixing” refers to a rearrangement pattern involving multiple steps between the ancestral genome and the descendent genome; first, chromosomal fusion, followed by one or more rearrangements that cause the syntenic blocks to interweave. For example, a single chromosome might contain a contiguous stretch of DNA encoding genes A, Z, X, B, Y, and C, in that order.

**Fig 3:**
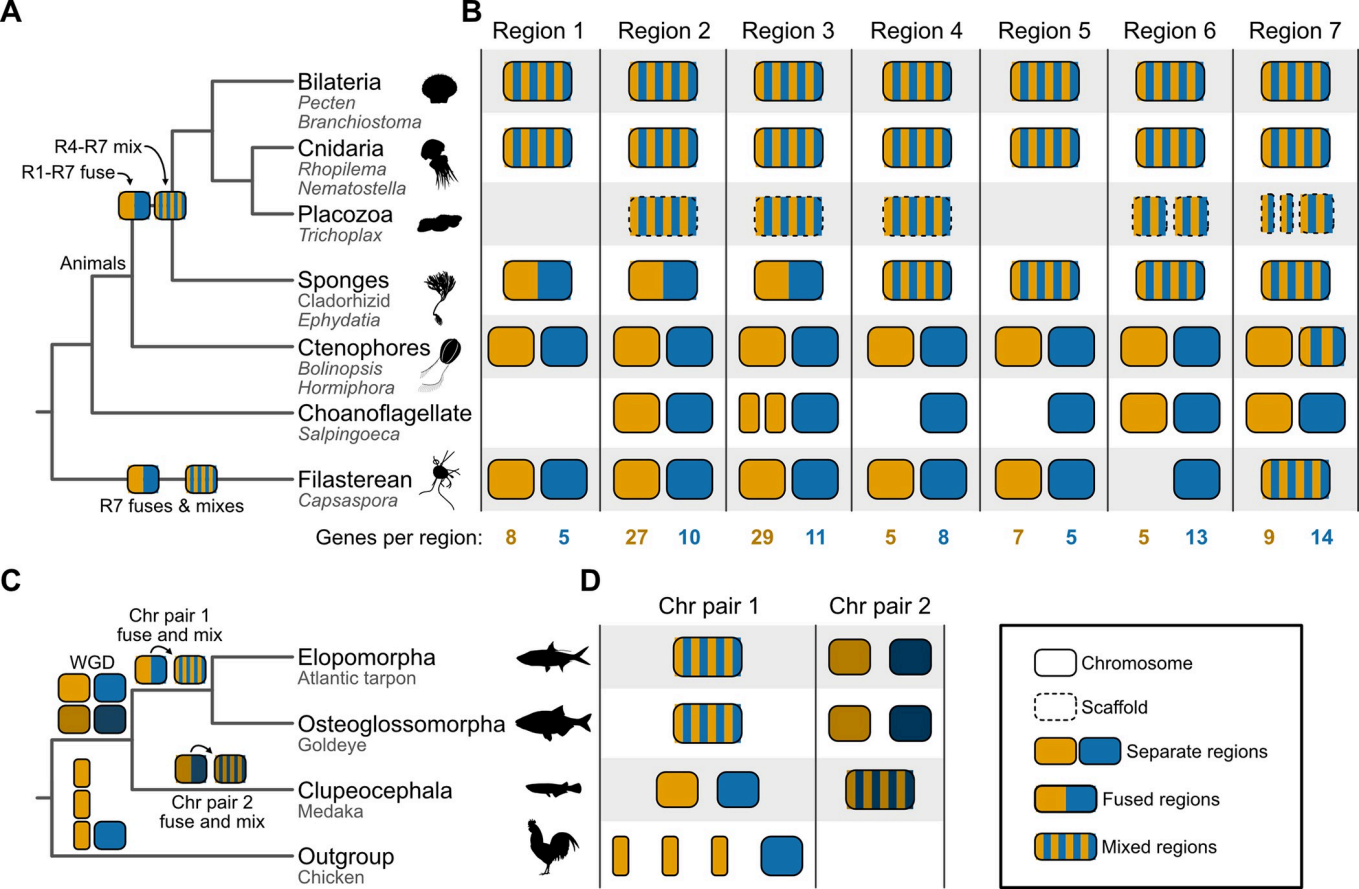
Summary depictions of syntenies supporting the ctenophore-first and EO-sister hypotheses. (**A**) Inferred phylogeny of animal and outgroup taxa used to examine the root of the animal tree. Under the ctenophore-first hypothesis, regions 1–7 each resulted from fusion events between 2 distinct chromosomes. The syntenic block depicted in orange for region 3 underwent a fission event in the choanoflagellate lineage, resulting in 2 chromosomes. Regions 4–7 underwent subsequent mixing events. Underneath each higher-order lineage name, the names of representatives used in the study [9] are listed. For example, among Bilateria, species from the genera *Pecten* and *Branchiostoma* were included in the study. Note, only fusion and mixing events relevant to rooting the animal tree are depicted. (**B**) Patterns of synteny in 7 different regions most parsimoniously support the ctenophore-first hypothesis. Examination of these regions indicates that all underwent fusion events and 4 also underwent mixing events. Each region is abbreviated as “R” along the phylogeny (for example, R1 refers to region 1). The number of genes in each syntenic region is listed at the bottom of the panel. (**C**) Inferred phylogeny of the 3 teleost fish groups, including an outgroup taxon (the chicken). Cartoon summary drawings of chromosomes are included for representative species. Common names of these species are provided below the taxonomic names. Highly contiguous genome assemblies facilitated the detection of chromosome fusing and mixing events after a whole genome duplication event. Chr, chromosome. (**D**) Chromosomes observed in extant species are depicted as cartoon summaries. Duplicated chromosomes from a whole genome duplication event are darkened. Silhouette images were obtained from PhyloPic (https://www.phylopic.org) and are dedicated to the public domain; all credit goes to their respective contributors.

For reconstructing the animal tree of life, the codified matrix of fusion events was then used for phylogenetic inference. The transition probability of changing from a fusion-with-mixing state to another state (i.e., fusion or fission state) was inferred to be unlikely (Fig 2E). Bayesian analysis of this data matrix supported the ctenophore-first hypothesis, as did direct examination of fusions analyzed using parsimony [9]. Specifically, the ctenophore-first hypothesis was supported by 7 fusion events shared by bilaterians, cnidarians, and sponges, but that were missing from extant ctenophores and outgroup taxa. Four of these events occurred with mixing; under the sponge-first hypothesis, convergent fusions-with-mixing or precise reversions are required to explain these data. Thus, the absence of these fusions from ctenophores and outgroup taxa (except variation in region 7) was interpreted as evidence that ctenophores diverged from all other animals before the fusion and mixing events (Fig 3A). Region 7 may have independently undergone fusion and mixing events in the Filasterean lineage. An alternative but less likely scenario is that region 7 was already in a “mixed” state in the ancestor of all sampled taxa and subsequently underwent demixing and defusion events, followed by a complex pattern of fusion and mixing events.

Nonetheless, other findings from the synteny analysis contradict well-established evolutionary relationships. For example, despite phylogenomic analyses robustly supporting choanoflagellates as the closest living relatives of animals [78–82], animals shared more syntenic blocks with the filasterean than with the choanoflagellate (29 syntenic blocks compared to 20). There are also more unique syntenic blocks shared between the filasterean and animals than with the choanoflagellate (9 syntenic blocks compared to 2). The incongruence between the pattern of synteny conservation and prior findings from phylogenomics either suggests a previously undetected close evolutionary relationship between filastereans and animals or, more likely, a lineage-specific loss of synteny in choanoflagellates.

Indeed, some choanoflagellates have undergone unique, accelerated genome evolution. Specifically, the choanoflagellate *S*. *rosetta* (used in [9]) has experienced rapid gene family evolution compared with other choanoflagellates, resulting in a reduced gene repertoire relative to that of the last common ancestor of animals and choanoflagellates [83]. Accordingly, *S*. *rosetta* may not be the best representative of choanoflagellates for phylogenetics, highlighting the importance of expanded taxon sampling.

Similarly, unbiased phylogenetic analysis of fusion states did not recover the monophyly of Porifera, which contradicts more recent phylogenomic studies supporting the monophyly of the lineage [24,25,84]. Although some analyses support paraphyly among Porifera [85,86], the exemplar sponges in the study [9] belong to the class Demospongiae, which most analyses support as a monophyletic clade [87]. These observations call for caution in using syntenic blocks, especially when synteny has been lost.

#### Synteny and the evolutionary relationships among major groups of teleost fish

Early branching patterns in the teleost fish phylogeny were also recently reexamined [8] using a combination of expanded taxon sampling and analysis of syntenic blocks. Synteny was detected using the position of orthologous genes along chromosomes for every pairwise comparison of species. Phylogenetic analyses of the resulting macrosynteny and microsynteny data (Fig 2A and 2B)—wherein lack of syntenic conservation was used to measure distance—supported the EO-sister hypothesis. Using macrosyntenies, nearly 20% of breakpoints supported the EO-sister hypothesis, and using microsynteny data, the sister relationship between these lineages received full bootstrap support. Evidence of a single chromosome fusion event unique to slim-headed and bony-tongued fish and another unique to other teleosts corroborated the EO-sister hypothesis; specifically, after a whole genome duplication event along the stem lineage of teleosts, 1 chromosome pair fused among slim-headed and bony-tongued fish, whereas the other chromosome pair fused and mixed among other teleosts (Fig 3C and 3D).

In addition to synteny-based analyses, standard phylogenomic approaches based on sequence data were employed. Phylogenomic analyses and distributions of support frequencies based on analyses of single genes supported the EO-sister hypothesis (Fig 1K) [8]. Interestingly, this finding was not supported by previous studies examining single-gene support frequencies and ultraconserved elements under a maximum likelihood framework [88,89]. Thus, with this expanded set of taxa, the EO-sister hypothesis is supported by synteny analysis as well as by gene sequence concatenation and coalescence, pointing to the influence of expanded taxon sampling.

Analyzing data from more taxa generally improves phylogenetic inference, particularly among close relatives of phylogenetically unstable taxa [3,90,91]. For example, when represented by a single taxon, the placement of the Saccharomycotina family Ascoideaceae conflicted between 2 phylogenomic studies that likely did not suffer from insufficient locus sampling [92,93]. However, expanded sampling of genomes from 3 Ascoideaceae and close relatives robustly supported 1 hypothesis [94]. Additional analyses suggested that increased taxon sampling resulted in improved model fit and greater phylogenetic stability of focal lineages. These studies demonstrate how additional taxon sampling can improve phylogenetic inference. Moreover, the benefits of high-quality, chromosome-scale genome assemblies are multifold. For example, standard phylogenomic analyses have benefitted from synteny data to improve orthology predictions, and multiple data types, such as patterns of macrosynteny and microsynteny, provide additional lines of evidence for phylogenomic inquiry [95].

### Toward high-quality synteny-based tree of life reconstructions

**Fig 4:**
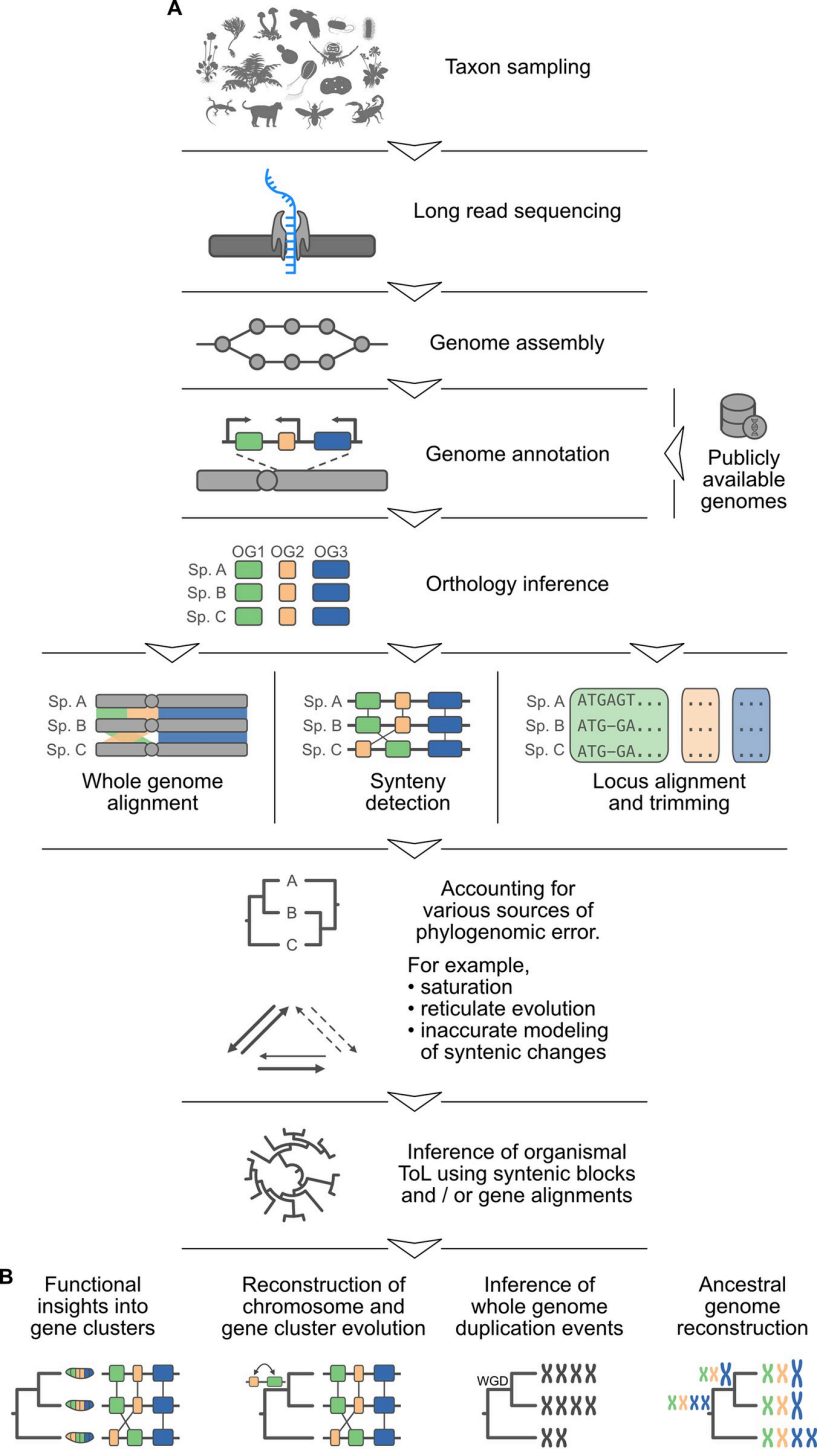
A roadmap of challenges and opportunities for synteny-based phylogenomics. (**A**) A high-level summary of steps toward best practices in synteny-based phylogenomics. Limitations in resource availability (computational power and researcher time) dictate that each project begins with a selection of taxa that are most relevant to the phylogenetic question at hand. For those taxa that lack high-quality genome assemblies, it will be necessary to sequence each genome (using long-read sequencing technology) and assemble the reads. In other cases, previously sequenced and assembled genomes may be publicly available. In either case, the next step is to annotate the genes in all selected genomes using a single high-quality annotation method. Comparisons among the gene complements of each organism should then be used to identify gene orthologs (orthologous loci are depicted in green, yellow, and blue). Orthologs can then be used in whole genome alignment and synteny detection. In addition, alignments of orthologs can be trimmed, assembled into multiple sequence alignments, and used for traditional phylogenenomics. After accounting for various sources of error, synteny blocks and multiple sequence alignments can be used to infer the topology of the tree of life. Note that obstacles in one step may be overcome by backtracking in the roadmap; for example, insufficient genome assembly completeness may benefit from additional genome sequencing. (**B**) Synteny data and organismal histories can be used for numerous research opportunities, including a better understanding of gene cluster function and evolution, reconstructing chromosome evolution, and inferring whole genome duplication events and ancestral genomes. For functional insights into gene clusters, fly embryos are depicted alongside gene clusters indicating how gene cluster organization may influence fly development. Silhouette images were obtained from PhyloPic (https://www.phylopic.org) and are dedicated to the public domain. Additional icons were obtained from bioicons (https://bioicons.com) and are available according to the CC-BY 4.0 license. Credit for silhouette images and icons goes to their respective contributors.

As highly contiguous genome assemblies become more commonplace, our understanding of synteny as a phylogenomic marker will mature. Here, we provide a roadmap of research opportunities and identify challenges that will shape the use of synteny as a phylogenomic character (Fig 4A).

Considerations for inferring synteny-based phylogenies.

#### Taxon sampling/selection

Taxon sampling influences numerous downstream steps, such as orthology inference. Generally, the more taxa sampled, the better [3,90]. Selection of outgroup taxa can also influence phylogenomic inference; for example, the root of the animal tree is heavily influenced by the taxa selected [29]. Therefore, outgroup taxa should be carefully selected. Fortunately, there are a growing number of chromosome-level or highly contiguous genome assemblies that are publicly available for downloading and analysis. However, representatives from undersampled lineages may require genome sequencing. Thus, taxon sampling should be guided by the phylogenetic question at hand. For example, determining evolutionary relationships among vertebrates does not require taxon sampling among fungi; in fact, poor taxon sampling of distantly related taxa may introduce long branches and contribute to long-branch attraction artifacts [96,97].

#### Long-read sequencing and chromosomal conformation analyses

Much like traditional phylogenomics using collections of multiple sequence alignments, synteny-based phylogenomics starts with data acquisition. However, unlike multiple sequence alignment-based phylogenomics, high-quality genomes (ideally assembled accurately from telomere-to-telomere on all chromosomes) are necessary. The state of the art for genome assembly requires long-read sequencing (e.g., using Oxford Nanopore or PacBio) [98,99], which, in turn, requires acquisition of high-molecular weight DNA from each organism to be sequenced. For more complex genomes, chromosomal interactions detected from Hi-C analyses will help provide additional lines of evidence for subsequent steps, namely, genome assembly [100].

#### Genome assembly

With long-read sequences and chromosomal conformation data in hand, the next step for synteny-based phylogenomics is to generate an accurate and precise genome for each species to be analyzed. Poor genome assembly quality can be a source of error when detecting synteny [101] and, in turn, introduce errors in synteny-based phylogenomics. While there is no broadly accepted definition of a “high-quality” assembly, researchers should consider 3 important metrics: completeness, contiguity, and accuracy. Completeness can be assessed by comparing inferred gene content with expectations from transcriptome sequences and the presence/absence of nearly-universal single-copy orthologs [102]. Incomplete genomes may be difficult to incorporate into synteny-based phylogenomics and may necessitate further efforts to improve the original genome assembly. When highly contiguous genomes are difficult to achieve, macrosyntenic blocks that are broken up across several scaffolds should be removed from the data matrix. Alternatively, microsyntenies may be more appropriate to use because they are more likely to be preserved, even in a discontiguous genome assembly. Examining assembly accuracy is difficult without physical mapping data from, for example, fluorescence in situ hybridization or optical maps [103]. However, these data can be useful, not only to validate, but also to improve genome assembly quality, even helping achieve near-complete genomes [103]. Of note, other measures of assembly quality, such as degree of contamination, should be taken into account, particularly when loss of synteny is inferred.

#### Genome annotation

To detect syntenic blocks across the resulting set of genomes, the relative positions of orthologous genes are often used [72,76]. Thus, phylogeneticists must predict gene boundaries accurately to prevent, for example, erroneously combining 2 genes into a single gene model or missing genes entirely (Fig 4A). Many phylogenomic studies rely on the outputs of genomes annotated using different methods, but recent studies have shown that the outputs of different gene annotation methods can vary substantially [104]. A troubling result of comparing genomes annotated using different annotation methods is the artifactual inflation of the number of unique or lineage-specific genes [104]. Therefore, a single high-quality annotation method trained on the individual organism, or methods that combine the results from multiple gene annotation algorithms, like EVidenceModeler [105], may prove helpful. Moreover, incorporating transcriptomic reads will help refine and provide evidence for gene boundary predictions [106].

#### Orthology inference

The resulting gene predictions are subsequently used to infer orthologous relationships among genes (Fig 4A). Orthology relationships are inferred using all-versus-all sequence similarity information [107]. Researchers face several challenges during orthology inference, stemming from both analytical and biological sources of error [3,108]. Analytical errors may stem from genes that are absent from annotation predictions but that are genuinely encoded in the organism’s genome. Other sources of incongruence between the evolutionary history of loci and the species may stem from complex evolutionary histories, such as gene duplication and loss, convergence, or saturation [3,109].

Alternatively, whole-genome alignment methods, like Progressive Cactus and SibekliaZ [110,111], may overcome potential errors stemming from gene annotation errors. One major innovation offered by Progressive Cactus is that it allows reference-free multiple genome alignment (ameliorating reference-based bias) and detecting multicopy orthology relationships, rather than only single-copy orthology [111]. Furthermore, Progressive Cactus can also handle large datasets, such as 600 or more animal genomes.

### Establishing best practices in synteny detection

Typically, the distributions of gene orthologs along chromosomes in different species are used to detect potential syntenic blocks. Therefore, differences in the quality of ortholog prediction and in the density of syntenic orthologs detected should profoundly shape the accuracy of syntenic block detection. Both factors—accuracy of ortholog detection and density of syntenic orthologs—will likely drop off when comparing genomes separated by long evolutionary time scales.

Care must be taken, therefore, in the selection of software and analysis parameters [101]. Two key parameters are the minimum number and density of genes necessary to define orthologous syntenic blocks. Higher thresholds are expected to result in more conservative estimates of syntenic blocks (i.e., fewer false positives), but at the cost of potentially having a smaller number of syntenic blocks to analyze. Several software packages facilitate synteny detection, including MCScanX, SynChro, and syntenet [61–63]. Notably, each employs different methodology; for example, SynChro identifies pairwise syntenies using reciprocal best BLAST hits of protein sequence similarity, whereas MCScanX detects synteny blocks across 2 or more genomes [61,62]. MCScanX also provides additional utilities to further classify syntenic blocks based on putative evolutionary origins, such as those originating from whole genome duplication events or tandem duplication. Although these algorithms vary in efficacy, genome discontiguity appears to be a major driver of error, underscoring the importance of obtaining highly contiguous genome assemblies [101].

To determine how much of the genome is captured during synteny detection, syntenic coverage can be calculated [101]. Syntenic coverage may differ between genomes due to biological phenomena such as genome size, content variation, or analytical factors that can relax the definition of a syntenic block; thus, it will be important to report syntenic coverage for individual genomes as well as summary statistics across them. Ideally, syntenic coverage will be high and cover nearly the entire genome for closely related organisms. However, syntenic coverage may be reduced depending on the threshold applied for detecting synteny, the rate of evolution among chromosomes, the rate of evolution of local gene order, and the evolutionary distance between species analyzed.

### Accounting for sources of phylogenomic error/noise

Diverse factors can lead to erroneous species tree inference. Although these are well studied in analyses of multiple sequence alignments [3,108,112], they are underexplored for synteny-based phylogenomics. Here, we discuss potential sources of error/noise for synteny analysis and methods for taking them into account.

#### Saturation

In nucleotide and amino acid sequence evolution, when multiple, unobservable substitutions occur, the precise stepwise evolutionary history is difficult to trace; this phenomenon is described as “saturation.” Saturation may also occur during synteny evolution, whereby multiple sequential rearrangements may interfere with tracing the step-wise evolution of syntenic blocks. To overcome saturation, one solution may be to purge data matrices of rapidly evolving syntenic blocks, wherein the evolutionary history may be harder to trace.

#### Incomplete lineage sorting

The random sorting of ancestral polymorphisms can lead to genealogies that differ from the species tree, especially during rapid radiation events [6,113]. Incomplete lineage sorting among structural variants may also be a source of synteny-based phylogenomic noise. Incomplete lineage sorting among gene trees is particularly prevalent during radiation events and in large populations [113,114]. Given that genome rearrangement can occur rapidly in a population [115,116], it raises the possibility that some structural variants may coalesce before a speciation event, i.e., be subject to incomplete lineage sorting. Determining the prevalence (if any) of incomplete lineage sorting among structural variants will elucidate if incomplete lineage sorting is a source of incongruence.

#### Reticulate evolution

Reticulate evolution refers to nonvertical inheritance of loci, resulting in loci with an evolutionary history that deviates from a strictly bifurcating tree model, such as horizontal gene transfer and introgression/hybridization [117–119]. This issue will have varying influences across different lineages; for example, horizontal gene transfer occurs more frequently among Bacteria and Archaea than many eukaryotic lineages [120,121]. Similarly, hybridization is common among plant lineages [122–124] and has also been observed in other lineages, including animals and fungi [118,125–127].

The nonvertical acquisition of loci may interfere with the detection of otherwise conserved syntenic regions [128]. In the case of horizontal gene transfer, synteny analysis would suggest an erroneous phylogenetic placement of a lineage; for example, synteny analysis of the horizontally acquired bacterial siderophore gene cluster in yeast [129] would suggest a close affinity between yeast and Bacteria, a hypothesis that is incontrovertibly refuted. Loci with signatures of horizontal gene transfer can be pruned from a data matrix. However, in some cases, horizontally acquired loci that undergo vertical inheritance may be helpful markers for synteny-based phylogenomics [130].

#### Modeling syntenic changes

In standard molecular phylogenetics, substitution models approximate the evolutionary process of transitions between character states. These models vary in complexity and ability to capture biological reality [131–133]. Analogous substitution models for syntenic data have yet, to our knowledge, to be developed. However, structural variants can segregate among human populations [44] and recent developments of reference-free pangenomes may help facilitate their detection and illuminate their evolutionary dynamics [134], paving the way for creating models that capture exchange rates between syntenic states. The empirical determination of best practices for model selection will be important for future studies. Assuming overfitting is not an issue, highly parameterized models may be appropriate for synteny-based tree inference.

#### Other potential sources of error

Several other sources of error may come into play. For example, although few examples of convergent evolution in genome structure are known [135–137], they nonetheless demonstrate how independent rearrangements that result in the same structure could contribute to noise in synteny-based phylogenomics. Specifically, the currently accepted evolutionary relationships among the major rodent clades of Hystricomorpha (e.g., capybaras and naked-mole rats), Sciuromorpha (e.g., squirrels and marmots), and Myomorpha (e.g., rats and mice) indicate that Hystricomorpha diverged first and that Sciuromorpha and Myomorpha are sister lineages [136]. However, independent splitting events in the ortholog of human 3p21.31 in the Hystricomorpha (e.g., capybaras) and Sciuromorpha (e.g., squirrels) lineages would incorrectly suggest a sister relationship between each lineage [136]. Other sources of error may include an underpowered number of syntenic blocks and intraspecies heterogeneity in karyotype and chromosome structure due to, for example, Robertsonian translocations and copy number variants [77,115].

For phylogenomic analyses based on collections of multiple sequence alignments, researchers have demonstrated that not all loci have equal phylogenetic information. For example, genes displaying a clock-like pattern of evolution have often been favored for divergence-time analysis [138–140]. Measures have been developed to quantify the information encoded in multiple sequence alignments and phylogenetic trees inferred from them. Fortunately, some methods may be easily adapted to synteny data. For example, treeness [141] may help identify syntenic blocks with robust phylogenetic signal. Similarly, rogue taxa can be pruned from a data matrix [91]. Developing methods to measure the phylogenetic informativeness of different syntenic blocks will help increase signal-to-noise ratios among datasets and aid in refining their usage and interpretation within phylogenomic analyses.

### Research opportunities using synteny data and species trees

Developing best practices for accurate synteny-based phylogenomics will help address current gaps in our understanding of genome evolution. For example, not only will synteny-based phylogenomics offer a new perspective for tree of life reconstructions, but the underlying synteny data may help provide functional insights into gene clusters (Fig 4B). Synteny-based phylogenomics will also help trace the evolution of chromosomes and gene clusters along phylogenies. Such reconstructions will help identify whole genome duplication events, which have been of longstanding interest to biologists because they provide fodder for molecular innovation, such as functional divergence of the resultant ohnologs [142,143].

Synteny-based phylogenomics may also facilitate ancestral genome reconstruction, potentially enabling (near) reference-level assemblies given sufficient sequenced and assembled genomes from extant species (Fig 4B). Accurate reconstructions of ancestral genomes, coupled with ancient DNA sequencing, may help resurrect the genomes of extinct lineages. More broadly, a complete understanding of synteny evolution across time and species will contribute to a unified theory of genome architecture evolution.

While these opportunities present only a few exciting research prospects, phylogeneticists must first prioritize evaluating the efficacy of synteny-based phylogenomics for reconstructing ancient and recent divergences, spanning species and populations.

## Conclusions

Improvements in genome sequencing, assembly, and annotation have revolutionized the quest to reconstruct the tree of life. With cutting-edge technologies and algorithms that enable the inference of highly contiguous genomes, synteny has reemerged as a powerful character for tree of life inquiries. Two studies tackling longstanding debates in animal phylogeny serve as notable case studies for demonstrating the potential utility and caveats of using synteny to reconstruct life’s history [8,9]. These studies mark a new chapter, in which synteny-based phylogenomics promises to bring fresh insights, albeit after a series of technical challenges have been overcome. Tackling these challenges head-on will help shape best practices and deepen our understanding of synteny-based phylogenomics.

It is unlikely that Sturtevant and Dobzhansky, pioneers of their time in the 1930s, could have foreseen the far-reaching implications of their work on synteny as a phylogenetic marker. Nonetheless, their efforts have laid the groundwork for discoveries that continue to unfold today, nearly a century later, as technological advances enable the realization of their ambition. Uniting phylogenomics with comparisons of genome architecture in a whole-evidence approach promises to illuminate the detailed topology of the tree of life.
